# Supplementary material for: The effects of prolonged single night session of videogaming on sleep and declarative memory
Source: PLoS One. 2019 Nov 21;14(11):e0224893. doi: 10.1371/journal.pone.0224893 (PMC6874076; doi:10.1371/journal.pone.0224893)
Supplement: Attachment — Submitted filename: Rewiev PONE.pdf [file pone.0224893.s002.pdf]

Dear authors,

It was a pleasure to review your work. Topic of video games, sleep and cognitive components is very beloved to me. I reviewed the paper very carefully.

Despite the countless criticisms and oppositions that are made to studies in this sector, in this work you have succeeded in bypassing them (for example the small sample, male subjects, length of exposure to video games, number of hours per night). This shows a good knowledge of the field literature. I also appreciated the statistical choice to use median and quartiles to facilitates comparison of our results to the literature.

However the paper needs further investigation.

1 - You used a violent video game (Global strike - Global Offensive) and a board game (Monopoly). Why didn't you use other kinds of video games? More information could be collected. In general video game is very activating (see Kovess-Masfety et al (2016). Is time spent playing video games associated with mental health, cognitive and social skills in young children?. *Social psychiatry and psychiatric epidemiology*, *51*(3), 349-357). While the board game is less activating (in fact the arousal of the BN is lower) and consequently you have received more complaints about 5 hs of playing a board game being too long. It is considered a boring activity, obviously sleep arises first. BN group could be a control group.

2 - Why did the subjects participate in both experimental conditions? In this way it was possible to create an expectation effect.

3 – In "Procedure" you say that "50% of the participants played the board game on the second weekend and the video game in the third weekend, the other half played in reverse sequence". Have you analyzed if the different sequences (VN-BN and BN-VN) gives different effects on variables? Compare the results of sleep variable, free recall, vigilance and sleepiness, melatonin, cortisol, etc… in the two groups that followed different sequences.

4 - It is true that 300 minutes of playing video games are many, but the exposure is only one. Specify in the paper that the effects occur following a short-term exposure. The title could be changed to "The effects of prolonged single night session of videogaming on sleep and declarative memory"

5 – In the first phrase of the paragraph "Discussion of Results", word "detrimental" is too strong. Replace with a more suitable term.

6 – Which Correct Words value is reported in the paper? Pre- or Post- sleep? This is unclear.

7 - Why didn't you use a non-verbal declarative memory test? For example a visual declarative memory test given that the activities performed were all visual. Other information could emerge (see Peracchia, S., & Curcio, G. (2018). Exposure to video games: effects on sleep and on post-sleep cognitive abilities. A sistematic review of experimental evidences. *Sleep Science*, *11*(4), 302.)

8 - Update the literature.
