# Supplementary material for: The effects of prolonged single night session of videogaming on sleep and declarative memory
Source: PLoS One. 2019 Nov 21;14(11):e0224893. doi: 10.1371/journal.pone.0224893 (PMC6874076; doi:10.1371/journal.pone.0224893)
Supplement: Attachment — Submitted filename: Response to Reviewers06.09.2019.docx [file pone.0224893.s003.docx]

PLOS ONE	Friday, 6^th^ September 2019

Dear Editor and Reviewers,

Thank you for your carefully composed reviews and the chance to revise the manuscript. It was a pleasure to read your comments. In the following text we respond to your suggestions point-by-point.

Journal Requirements:

1) *Please ensure that your manuscript meets PLOS ONE's style requirements, including those for file naming.*

Response: We revised the layout so it hopefully meets PLOS ONE's style requirements.

2) *Please state in your methods section whether you obtained consent from parents or guardians of the minors (participants aged under 18) included in the study or whether the research ethics committee or IRB approved the lack of parent or guardian consent.*

Response: In case of participants aged under 18, the parents or guardians gave their consent. This information is added to the methods (l. 136-137).

3) *We note in your conclusion you indicate that you obtained 'surprisingly large' effects on video gaming on declarative memory. However, no effect sizes have been reported in the Results section. Please correct and/or clarify how you determined your effects were large.*

Response: We are sorry that this part is not clear in the Result section. Our conclusion refers to l. 228-231 and l. 246-252 in the Result section where the effect sizes are shown. We changed the Result part to make clear that these effects are related to the declarative memory.

4) *Thank you for stating the following in the Competing Interests section: [The authors have declared that no competing interests exist.]. We note that you received funding from a commercial source: Loewenstein Medical GmbH. Please provide an amended Competing Interests Statement that explicitly states this commercial funder, along with any other relevant declarations relating to employment, consultancy, patents, products in development, marketed products, etc. Within this Competing Interests Statement, please confirm that this does not alter your adherence to all PLOS ONE policies on sharing data and materials by including the following statement: "This does not alter our adherence to PLOS ONE policies on sharing data and materials.” (as detailed online in our guide for authors http://journals.plos.org/plosone/s/competing-interests).  If there are restrictions on sharing of data and/or materials, please state these. Please note that we cannot proceed with consideration of your article until this information has been declared. Please include your amended Competing Interests Statement within your cover letter. We will change the online submission form on your behalf.*

Response: We are sorry that we made an inexplicit declaration. This study was funded by the Löwenstein Foundation. This is a non-profit foundation that supports medical research, visual arts and people in need (https://www.loewensteinstiftung.de, http://addinter.service24.rlp.de/cgi-bin-inter/stiftung1.mbr/einzel?ID=837).

The foundation had no role in study design, data collection and analysis, decision to publish, or preparation of the manuscript and there were no payments to the authors. We included our statement within our cover letter and hope it is clear enough now.

5) *We note that you have stated that you will provide repository information for your data at acceptance. Should your manuscript be accepted for publication, we will hold it until you provide the relevant accession numbers or DOIs necessary to access your data. If you wish to make changes to your Data Availability statement, please describe these changes in your cover letter and we will update your Data Availability statement to reflect the information you provide.*

Response: The data underlying the results presented in the study are available from the sleep laboratory UKGM Marburg. We have compiled the relevant data and uploaded it as “Supporting information”.

Stefano Triberti, Ph.D., Academic Editor:

1) *I suggest Authors to (1) add a more specific limitations section with this and other limitations identified by Reviewers explicitly stated*

Response: More limitations have been included in the discussion (i.e. boring Monopoly/exciting Counter Strike: l. 320-322; no adjustment of the bed/wake times to the habitual sleep times and a quite short time in bed: l. 326-330)

2) *When referring to the experiment (text, tables, figures included), to not report the comparison between "video gaming and board gaming", but between "counter strike and monopoli", as these products are not necessarily representative of all the extremely rich gaming scenario.*

Response: We changed "video gaming“ into "Counter Strike: Global Offensive“ and “board gaming“ into "Monopoly“. This includes the tables, figures and most parts of the text.

3) *About this: the two games used should be described in more detail, given that they are the only experimental operators.*

Response: A more detailed description is inserted to the methods (l. 161-167).

4) *Future research section could be improved as well, taking into account that comparison between different board/video games could potentially lead to opposite results.*

Response: We added this idea to the conclusion part (l. 412-415), as suggested.

Reviewer 1:

1) *You used a violent video game (Global strike - Global Offensive) and a board game (Monopoly). Why didn't you use other kinds of video games? Video game is very activating (see Kovess-Masfety et al (2016). Is time spent playing video games associated with mental health, cognitive and social skills in young children?). While the board game is less activating (the arousal of the BN is lower) and consequently you have received more complaints about 5 hs of playing a board game being too long. It is considered a boring activity, obviously sleep arises first.*

Response: "Counter Strike: Global Offensive" is one of the most popular video games among adolescents and all of our participants had at least some experience with the game. We used this violent video game because of its high topicality, the possibility of team playing including fighting against each other. In our small sample, we wanted to maximize potential effects by playing a violent game for a very long time (five hours). Since 2018, after our study measurements, the video game "Fortnite" has become the most popular video game among 12 – 19 years old German adolescents (Medienpädagogischer Forschungsverbund Südwest. JIM 2018 Jugend, Information, (Multi-)Media: Basisstudie zum Medienumgang 12- bis 19-Jähriger in Deutschland 2018). Nowadays this game could be an alternative video game condition.

In line with the Academic Editor Mr. Triberti we added a paragraph about the comparison of the rather boring “Monopoly” and the more exciting computer game (l. 320-322; l. 412-415). In general, we consciously intermingled the aspect “exciting” with video gaming and “less exciting” with board gaming because we wanted to maximize effects. The drawback that inevitably goes along with that decision has been emphasized in the new version of the manuscript. Another not so scientific reason for choosing monopoly were the reports of the more senior co-authors (Koehler, Cassel) that they had spent considerable time with playing Monopoly as adolescents.

2) *Why did the subjects participate in both experimental conditions? In this way it was possible to create an expectation effect.*

Response: The first weekend was just a habituation phase with no gaming conditions. The adolescents participated in a reverse sequence model so 50 % played "Counter Strike: Global Offensive" at the second weekend and "Monopoly" at the third weekend. The other 50 % played "Monopoly" first and then "Counter Strike: Global Offensive". Interindividual sleep can vary widely. With our small sample size of 20 (including two drop outs) participants we wanted to prevent a high interindividual variation. While independent samples would have avoided the expectation effect, they would have introduced more interindividual variations requiring larger sample sizes which would have been very difficult to achieve.

3) *In “Procedure” you say that “50% of the participants played the board game on the second weekend and the video game in the third weekend, the other half played in reverse sequence”. Have you analyzed if the different sequences (VN-BN and BN-VN) gives different effects on variables? Compare the results of variable in the two groups that followed different sequences.*

Response: Thank you for this suggestion. We have compared the results in the two different groups. Sequence 1: Counter Strike - Monopoly; Sequence 2: Monopoly - Counter Strike. Unfortunately, the two drop outs of the study were both in the sequence 2 group, so there are just eight data sets in sequence 2 and ten data sets in sequence1 (l. 168-172 in the manuscript).

There was a sequence effect in the following variables (CS=VN, MP=BN):

Sleep variables:

N1-Duration MP: Seq 1 = 35,35 ±13,09; Seq 2 = 20,38 ± 15,42; p = .034

N3-Duration CS: Seq 1 = 90,85 ± 32,38; Seq 2 = 124,32 ± 32,82; p = .027

Pupillographic sleepiness test:

PUI CS: Seq 1: 4,45 ± 1,97; Seq 2: 7,59 ± 1,95; p = .004

VigiMar:

MRT CS: Seq 1: 1,17 ± 0,62; Seq 2: 3,24 ± 2,34; p = .027

Cortisol:

Cortisol 11.37 PM MP: Seq 1: 0,69 ± 0,25; Seq 2: 0,35 ± 0,20; p = .003

Cortisol 02.10 AM MP: Seq 1 0,69 ± 0,14; Seq 2: 0,44 ± 0,33; p = .016

To sum up, we can identify the following points:

1. Those who played Counter Strike in the first night

- had less deep sleep after the videogame but more light sleep after Monopoly
- were more alert on the next morning after Counter Strike during the VigiMar and PST

Despite of the first habituation night, the combination of Counter Strike and the first night with measurements could have been more exciting than Monopoly and measurements.

2. Those who played Counter Strike in the first night

- had higher cortisol levels directly after and in the night after Monopoly

Perhaps the adolescents were more annoyed of the board game. They have already played the more entertaining computer game on the other weekend. In regards to the group structure of four or five adolescents they were glad and excited that the measurements come to an end so they pushed each other for the last time.

Admittedly, these findings and interpretations are rather vague so we did not include them into the manuscript. We think they would rather distract the reader than give additional good structured information.

4) *It is true that 300 minutes of playing video games are many, but the exposure is only one. Specify in the paper that the effects occur following a short-term exposure. The title could be changed to "The effects of prolonged single night session of videogaming on sleep and declarative memory"*

Response: That is an excellent point. Thank you! We changed the title as suggested.

5) *In the first phrase of the paragraph “Discussion of Results”, word “detrimental” is too strong. Replace with a more suitable term.*

Response: We replaced the word with "negative".

6) *Which Correct Words value is reported in the paper? Pre- or Post- sleep? This is unclear.*

Response: We are sorry that this point is not clear. It depends on different parts in the paper. One of our main questions was if there is a difference in declarative memory between "Counter Strike" and "Monopoly" on the next morning, so this means post-sleep (results in l. 228-231)

In addition, we compared differences in declarative memory performance directly after the "Counter Strike"/ "Monopoly" stimulation, so this means pre-sleep (results in l. 246-252). We added "pre- and post-sleep" in some parts of the paper (l. 251; l. 341; l. 115; l. 231)

7) *Why didn't you use a non-verbal declarative memory test? For example a visual declarative memory test. Other information could emerge (see Peracchia, S., & Curcio, G. (2018). Exposure to video games: effects on sleep and on post-sleep cognitive abilities. A sistematic review of experimental evidences)*

Response: The most important point for the decision for the memory test was the possibility for repeating testing. The VLMT has got three comparable versions for every night. A non-verbal test could be a good alternative but it was a conscious decision to choose a verbal test because we did not want to use a screen at the board game night.

8) *Update the literature.*

Response: We updated and checked the literature. Thank you for your suggestions.

Reviewer 2:

1) *How was the VLMT administered? The text states that the list was memorized through five repetitions, but do not state whether words were presented one at a time, all together, etc., how much time participants were given to study the list, how much time passed in between repetitions, whether it was a computerized task, etc. Nor do the authors mention how much time participants were given for the free recall of the word list. It is not clear what is meant by “the recall was preannounced in each repetition” –does this mean participants were explicitly instructed to memorize the words?*

Response: Sorry, this is not described clearly in the paper. Every participant knew that he would have to repeat the words.

The investigator read out all 15 words, then the participant had to reproduce all the memorized words. After that, the investigator read out all the words again and the participants repeated. In sum this happened five times. Afterwards a different list of 15 words was read out and the participant had to repeat them. Then they played Counter Strike or Monopoly. After the game (pre-sleep) there was another recall without a repetition of the words. On the next morning (post-sleep) the participants had to repeat the words again without a preceding repetition. There was no exact time limit for the recall but after approximately a minute the recall was stopped.

We added more information about the VLMT in the methods (l. 151-158).

2) *For the 5 hours of gaming, were participants gaming continuously without breaks (other than the scheduled break at 8:55 to collect saliva)? It is also unclear whether the PCs were the sole source of light while playing video games or if there was additional ambient room lighting. Was the 20 lux of ambient lighting during the board game also measured at eye level? I am also curious how 20 lux was selected for the ambient lighting during the board game, as it is quite dim compared to ordinary room lighting. Finally, what lux was the room during the sleep period?*

Response: There were no additional breaks during the stimulation. In fact, the participants were offered some food and drinks but they were told to play while eating.

There was additional room light while playing video games and the light intensity was measured at eye level in both conditions. The eye-screen-distance was about 30 cm. We chose a more ambient light during the board game because we wanted to reproduce a quiet board game evening at home like it may have typically happened 40 years ago. We added more information about that in the new version of the manuscript (l. 176; l. 373).

3) *Were habitual bed/wake times taken into account during screening? Especially because sleep is one of the primary outcome measures, it is important to note whether the lab-scheduled sleep opportunities are at similar times to the participants’ habitual sleep times. If participants are attempting to sleep at times very different from their normal time, their sleep may be impacted independent of gaming condition.*

Response: Unfortunately, there has been no adjustment of the personal habitual bed/wake times. Every night had a tight schedule in the sleep laboratory. The participants played the games for five hours together so it was not possible to create a timetable referring to everybody’s bed times. But you are definitely right. We added this topic to the discussion part (l. 326-330).

4) *How was the timing of the saliva samples chosen? Also, it would be helpful to report the average time and standard deviation of the fourth nocturnal saliva sample, so that readers are aware of the variability of this sample time.*

Response: The aim of the saliva samples was to show the time course of the two hormones. The samples were taken before, in the middle and at the end of the game. To see the progression, the fourth samples was taken in the night. The extraction should cause the least possible disturbance so a time was chosen when less REM or slow wave sleep was expected (having in mind that the typical sleep cycle lasts 70-100 minutes). The mean and standard deviation of the fourth saliva sample time is 02.10 AM ± 19 minutes. We added this to the paper. The paper was not submitted with the actual version of table 3 and 4. We are sorry for that and changed it. In a former version, there was a little excel fault with regard to the sample times.

5) *Why was the VigiMar test used to assess vigilance instead of the more commonly used Psychomotor Vigilance Test (PVT)? And given that the VigiMar is a very long test, were there any differences in time-on-task effects in the two conditions?*

Response: The VigiMar was developed in Marburg where the study was conducted. The employees of the sleep laboratory know the test very well so it was easy to use it. In addition to that, we wanted to investigate a long-term vigilance test. The VigiMar is frequently used in Germany and described in a standard textbook of the German Sleep Society about methods in sleep medicine (Kompendium Schlafmedizin).

We haven´t included any data about the time-on-task effect into our data set yet so at this point we cannot compare this effect in the two conditions. Generally, it´s possible to adopt additional information into our digital data set but therefore another analysis of our analogue raw data set is necessary. In effect, it means that we have to go back to paper source data and to check it with our quality assurance as we´ve done with the rest of our data. For this process more time would be needed. Until now, we haven´t paid special attention to the time-on-task effect because we thought it would be rather slight but generally an analysis could be supplemented if a future revision is required.

6) *The authors report significantly lower melatonin levels after video gaming compared to board games (pg 10) and state that this “indicates a phase delay of melatonin secretion”. While this clearly indicates melatonin suppression, and it is possible that evening video games could have caused a phase delay, the current experimental design does not allow them to assess whether there has actually been a phase shift. Similar on pg 14, line 360, the data show melatonin suppression but not necessarily a phase delay.*

Response: You are right that the design does not allow to state a phase delay. We modified the wording accordingly (l. 272; l. 377).

7) *Although the authors note that participants were scheduled to 7 hours time in bed, which is close to the average for German adolescents, they also report on page 5 that their participants reported sleeping on average 7.65 hrs in bed during the week and 9.02 hrs on the weekend. Thus, it is likely that they were either somewhat sleep restricting the participants, or at the very least scheduling wake times significantly different from the participants’ habitual wake. This should be mentioned in the discussion of scheduled time in bed.*

Response: This is a limitation of the experimental design and has been added to the discussion of the methods (l. 326-330).

8) *Minor typographical errors in the text:*

*a. Use consistent tense throughout the manuscript. E.g. on pg 2, line 28, change “does” to “did”; pg 14 line 355, change “seems” to “seemed”, etc.*

*b. Pg 5 in the ethics statement, it is not clear what “All participants were tired and informed…” is intended to say.*

*c. Pg 6, line 172 “brake” should be changed to “break”*

*d. Pg 15, line 393 “extend” should be changed to “extent”*

Response: Thank you for your excellent and very thorough review. We have corrected the errors.
