# Supplementary material for: The effects of prolonged single night session of videogaming on sleep and declarative memory
Source: PLoS One. 2019 Nov 21;14(11):e0224893. doi: 10.1371/journal.pone.0224893 (PMC6874076; doi:10.1371/journal.pone.0224893)
Supplement: Attachment — Submitted filename: Response to Reviewers.docx [file pone.0224893.s004.docx]

PLOS ONE	Friday,18^th^ October 2019

Dear Dr. Triberti, Dr. Peracchia and Reviewer 2,

Thank you for your reviews and the chance to revise the manuscript for a second time. First of all, please accept our sincere apologies for addressing you as Mr instead of the correct form Dr. In the following text we respond to your suggestions point-by-point again.

Dr. Stefano Triberti:

1) *Moreover, it had been asked according to journal requirements to add effect sizes, especially because the effect is deemed "surprisingly large" in conclusion. I was not able to find the effect sizes at the lines and tables Authors report in the response, but only means and p values. These should be added. I also suggest to modify those lines in conclusion; first, they seem unnecessary to comment on Authors' results, second they would be inappropriate unless the effect size is extremely high taking into consideration previous literature on the topic.*

Unfortunately, we have misunderstood your last suggestion for the first revision. You are right that no measures of effect sizes have been shown until now. We have calculated effect sizes for all statistically significant tests. We have added these to the tables and changed the text of the manuscript (i.e. l. 222-224, l. 235, l. 348-349, l. 412-414)

According to Cohen, all the reported r-values show a medium effect size for the statistically significant tests (r-values of 0.10, 0.30, and 0.50 to demarcate small, medium and large effects). Recent studies remark that Cohen's correlation guidelines are too exigent. They suggest to consider correlations of 0.10, 0.20 and 0.30 as relatively small, typical (medium) and relatively large for individual differences researchers. According to Gignac, our results show a large effect. (Gignac, Gilles E.; Szodorai, Eva T. (2016): Effect size guidelines for individual differences researchers. In: *Personality and Individual Differences* 102, S. 74–78. DOI: 10.1016/j.paid.2016.06.069.)

In our manuscript we used the established and rather conservative evaluation of Cohen's guidelines.

Dr. Sara Peracchia:

Thank you very much for your well composed review. We appreciate your positive decision.

Reviewer 2:

1) *I appreciate the opportunity to review the revised manuscript, and thank the authors for addressing the comments. My only remaining concern is that the revised description of the VLMT is still somewhat unclear. If the VLMT has been published previously, perhaps the authors could include a citation/reference with a more detailed description of the task? The parts I still found confusing are as follows:*

*Pg 6, L155 “…this list was read out by the investigator and reproduced by the participants for five times each”*

*It is unclear to me whether this means participants are verbally repeating the words as the investigator reads each word aloud, or if participants are being asked to repeat the full list from memory after the investigator has finished reading all 15 words, etc. From the comments to the reviewers, it sounds as though participants are being asked to freely recall all 15 words immediately after the investigator reads the full list, in which case it would be important to report whether participants received any feedback if they made errors or prompting if they were unable to successfully repeat all 15 words. The information about recall time should also be reported in the text.*

*Pg 6, L156-57 “…then another list with fifteen words was read to the participants who had to reproduce them”*

*Please clarify the purpose of this second list of words-- is it for masking/interference, or are participants tested on this list as well?*

We are really sorry that this part is still unclear. The participants were asked to recall the words after the investigator has finished reading all 15 words. The second list was just a distractor list. They were tested on this list as well, but only in one immediate recall which is not reported in the manuscript.

We have changed the description of the VLMT in the manuscript. Hopefully it is more understandable now:

“The VLMT consists of two lists, each with fifteen unrelated words (list A and list B). There is a parallel form for repeated testing. List A is read out by the investigator in five consecutive learning trials, each followed by an immediate free recall by the participants. This is followed by one round of reading and immediate recall of the distractor list. After the “Counter Strike: Global Offensive” resp. “Monopoly” stimulation (pre-sleep) and on Sunday morning (post-sleep) the participants were asked to recall the words from list A. There was no exact time limit for the recall, but it usually lasted less than one minute until subjects stated that they recalled no further words. There was no feedback about the number of correct recalled words.”

The VLMT represents a modified German version of the Rey Auditory Verbal Learning and Memory Test (RAVLT).

A version of the test has been published by Witt for example (Witt, JA., Coras, R., Becker, A.J. et al. Brain Struct Funct (2019) 224: 1599. https://doi.org/10.1007/s00429-019-01857-1).
